# Cortisol and adrenocorticotropic hormone concentrations in horses with systemic inflammatory response syndrome

**DOI:** 10.1111/jvim.15620

**Published:** 2019-09-12

**Authors:** Allison J. Stewart, Eileen Hackett, Francois‐Rene Bertin, Taylor J. Towns

**Affiliations:** ^1^ School of Veterinary Science The University of Queensland Gatton Queensland Australia; ^2^ Department of Clinical Sciences, College of Veterinary Medicine Auburn University Auburn Alabama; ^3^ Swedish University of Agricultural Sciences Uppsala Sweden; ^4^ College of Veterinary Medicine & Biomedical Sciences Colorado State University Fort Collins Colorado

**Keywords:** adrenal, CIRCI, colic, endocrinology, equine

## Abstract

**Background:**

Plasma adrenocorticotropic hormone (ACTH) and serum cortisol concentrations increase with illness‐associated stress. Dynamics of plasma ACTH and serum cortisol concentrations in adult horses with systemic illness are undocumented.

**Hypothesis/Objective:**

To determine whether ACTH and cortisol concentrations and the ACTH/cortisol ratio vary with survival, the presence of systemic inflammatory response syndrome (SIRS), or ischemic gastrointestinal lesions at admission, or throughout hospitalization.

**Animals:**

One hundred fifty‐one adult horses.

**Methods:**

Prospective study measuring serum cortisol and plasma ACTH at admission and on days 2, 4, and 6 of hospitalization. Horses were grouped by outcome (survival, SIRS status, number of SIRS criteria [SIRS score], SIRS severity group, and the presence of an ischemic lesion). Differences between groups and over time for ACTH, cortisol, and ACTH/cortisol ratio were investigated with a mixed effect model. Receiving operator characteristic curves and odds ratios were calculated for survival and ischemia.

**Results:**

In all groups, ACTH, cortisol, and ACTH/cortisol ratio significantly decreased over time (*P* < .0001). ACTH, cortisol, and ACTH/cortisol ratio were higher at admission in nonsurvivors, and ACTH and cortisol were higher in horses with ischemic lesions (*P* < .01). Horses with ACTH above reference interval at admission were 6.10 (2.73‐13.68 [95% confidence interval]) times less likely to survive (*P* < .0001). No significant difference in ACTH, cortisol, and ACTH/cortisol ratio between horses with different SIRS status, scores, or groups were detected, although nonsurvivors had a higher SIRS score (*P* < .0001).

**Conclusions and Clinical Importance:**

Pituitary and adrenal responses are altered in nonsurviving horses and those with an ischemic gastrointestinal lesion.

**Abbreviations**
ACTH: adrenocorticotropic hormone
CIRCI: critical illness‐related corticosteroid insufficiency
CRH: corticotrophin‐releasing hormone
HPA: hypothalamic‐pituitary‐adrenal gland
ICU: intensive care unit
OR: odds ratio
PPID: pituitary pars intermedia dysfunction
ROC: receiver operating characteristic
SIRS: systemic inflammatory response syndrome

## 1 INTRODUCTION

The hypothalamic‐pituitary‐adrenal (HPA) axis is the major neuroendocrine pathway involved in the homeostatic response to the stress of illness.^1^ Increasing circulating concentrations of corticotrophin‐releasing hormone (CRH), arginine vasopressin, ACTH, cortisol, and aldosterone are essential components of the stress response and are typically proportional to the magnitude of the injury or disease process.^1^ The systemic effects of cortisol are important in the homeostatic response to illness, including maintenance of blood pressure, provision of cellular nutrients, and moderation of inflammation.

Although cortisol and ACTH concentrations are expected to be high during acute illness, basal and ACTH‐stimulated cortisol concentrations lower than expected for the degree of illness have been identified in critically ill human patients and foals.^2^, ^3^, ^4^, ^5^ Although absolute hypoadrenocorticism is rare in intensive care unit (ICU) patients, relative adrenal insufficiency (RAI) is the most easily diagnosed cause of critical illness‐related corticosteroid insufficiency (CIRCI).^5^ This complex syndrome might also be because of inadequate CRH or ACTH production, or peripheral tissue resistance to cortisol.^5^ In human ICU patients, CIRCI is commonly caused by systemic inflammatory response syndrome (SIRS) and sepsis.^5^, ^6^ Inflammatory mediators released in response to illness can suppress CRH and ACTH secretion, thereby leading to impairment of cortisol synthesis.^5^, ^6^ Inflammatory cytokines such as tumor necrosis factor‐α, interleukin‐1, and interleukin‐6 can cause ACTH resistance and alter cortisol secretion and metabolism, which can result in a normal to high ACTH concentration and an inappropriately low cortisol concentration.^6^, ^7^ Inadequate cortisol production for the increased demands of critical illness can result in hypotension, hemodynamic instability, excessive inflammation, hypoglycemia, and nonspecific signs of depression, anorexia, and weakness.^5^

Cortisol has been measured in horses with colic and hospitalized foals,^3^, ^4^, ^8^, ^9^, ^10^, ^11^, ^12^ while ACTH has been measured in sick foals and a limited number of horses with colic.^3^, ^4^, ^8^, ^9^, ^10^, ^12^ Sparse information is available regarding endocrine function and dysfunction in sick adult horses throughout hospitalization,^13^, ^14^ but extensive adrenal gland hemorrhage, venous thrombosis, and adrenocortical necrosis are common findings in horses with ischemic gastrointestinal lesions at necropsy,^15^ providing a structural basis for development of RAI/CIRCI. Although incompletely understood, recognition and treatment of CIRCI in humans has improved survival rates, especially in the management of shock and sepsis.^16^, ^17^ A high ACTH/cortisol ratio was initially used in neonatal foals to diagnose hypothalamic‐adrenal dysregulation, indicating adrenal exhaustion, adrenal damage, or inadequate cortisol production and release in response to high pituitary output of ACTH.^3^, ^12^ In septic foals, nonsurvivors had significantly higher ACTH/cortisol ratios than did surviving foals, suggesting possible CIRCI in those nonsurvivors.^3^, ^12^ Relative adrenal insufficiency/CIRCI has been diagnosed by an inadequate cortisol response to exogenously administered ACTH or a low basal cortisol concentration in humans and foals.^5^, ^8^, ^9^

The overall objective was to describe ACTH and cortisol dynamics in spontaneously sick adult horses by evaluation of their associations with survival, SIRS, and the presence of ischemic gastrointestinal lesions. We hypothesized that cortisol and ACTH would be increased in proportion to illness severity, but a proportion would have HPA dysregulation with high ACTH/cortisol ratio. We expected that ACTH and cortisol concentrations would rapidly return to reference ranges in mildly ill horses, but might remain high in severely ill horses.

## 2 MATERIALS AND METHODS

### 2.1 Data collection

Horses (>1 year of age) presenting to the emergency service of 2 university teaching hospitals were recruited over 2 summers (mid‐May until mid‐August). Serum cortisol and plasma ACTH concentrations were measured at admission (before hospital administration of any medication) and on days 2, 4, and 6 if the horse was still hospitalized. A validated classification of SIRS was based on the presence of ≥2 of the following criteria: hypothermia (<37.0°C or 98.6°F), hyperthermia (>38.5°C or 101.3°F), tachypnea (respiratory rate > 20 breaths/min), tachycardia (heart rate > 52 beats/min), leukocytosis (white cell count >12 500/μL), leukopenia (white cell count <5000/μL), or the presence of > 10% band neutrophils.^18^ Horses were then assigned a SIRS score based on the number of SIRS criteria identified at admission (SIRS 0‐SIRS 4).^18^ Horses were then assigned into 3 groups: non‐SIRS (SIRS score 0 and 1); SIRS 2 (SIRS score 2 cases); and SIRS 3/SIRS 4 (SIRS 3 and 4 cases).^18^ Horses with clinical signs or a history associated with pituitary *pars intermedia* dysfunction (PPID) such as hypertrichosis, abnormal shedding, polyuria/polydipsia, or laminitis were excluded. Any case euthanized because of financial limitations without a poor prognosis was excluded.

Data recorded included signalment, physical examination findings, and routine bloodwork at admission (hematological and biochemical data), diagnosis, outcome, duration of hospitalization, and the presence of an ischemic gastrointestinal lesion for horses that underwent surgery or necropsy. Blood was collected by either venipuncture or an aseptically placed intravenous catheter and placed in a plain tube and a cold tube containing EDTA mixed with 0.3 mL of the protease inhibitor aprotinin (aprotinin lyophilized powder, USB Corp, Cleveland, OH) with final concentration: 500 kallikrein inactivator units/mL of blood. Blood was allowed to clot for 45 minutes at room temperature and centrifuged to collect serum, while plasma was centrifuged within 20 minutes. Samples were frozen at −80°C until assayed. Endogenous serum total cortisol and plasma ACTH concentrations (hereafter referred to as “cortisol” and “ACTH”) were measured in duplicate using radioimmunoassays (Coat‐A‐Count Cortisol In‐vitro Diagnostic Test Kit, Diagnostic Products Corporation, Los Angeles, CA; ACTH Immunoradiometric assay, Scantibodies Laboratory Inc, Santee, CA) previously validated in horses.^19^, ^20^ Intra‐ and interassay coefficients of variation were <15% and <19%, respectively, for ACTH, with upper limit of the laboratory interval <28 pg/mL.^20^, ^21^ The interassay and intra‐assay coefficients of variation for the cortisol assay ranged from 7.1% to 7.7% and from 6.1% to 8.1%, respectively.^19^ Laboratory reference range for cortisol concentration was 1.49‐6.45 μg/dL.^21^ All aspects of the study were approved by the universities’ Institutional Laboratory Animal Care and Use Committees.

### 2.2 Data analysis

Horses were categorized based on survival (discharged alive or not), the presence of SIRS (≥2 SIRS criteria), the number of SIRS criteria (SIRS score), SIRS group, and the presence of an ischemic lesion and compared. For continuous data, normality was assessed by a Shapiro‐Wilk normality test. No data followed a normal distribution; therefore, all were reported as median [range], as were ordinal data. Admission SIRS scores were compared between groups using a Mann‐Whitney test. Nominal data were reported as counts and percentage of horses in which the variable was documented. Counts were compared using either a Chi‐square test or a Fisher's exact test depending on expected counts. Odds ratios (OR) and 95% confidence intervals (CI) were calculated when appropriate. A linear mixed effect model determined the effect of survival, SIRS, SIRS score, SIRS group, or ischemia and time on ACTH, cortisol, and ACTH/Cortisol. Sidak's multiple comparisons test was used for post hoc analysis. A receiver operating characteristic (ROC) curve was plotted to analyze the prognostic value of a given variable (ACTH, cortisol, or ACTH/cortisol ratio) at admission for predicting survival, the presence of SIRS, or an ischemic lesion. The cutoff value was selected to optimize sensitivity and likelihood ratio. Commercially available statistical software was used (Prism, GraphPad Software, Inc. La Jolla, CA). *P* < .05 was considered statistically significant.

## 3 RESULTS

### 3.1 Animal population

One hundred fifty‐one horses were included and ranged from 1 to 34 years of age with a median age of 11 years. Fifty‐nine horses (39%) were female and 92 (61%) were male including 82 geldings (89% of males) and 10 stallions (11% of males). Breeds included Quarter Horses and associated breeds (60 horses, 40%), Tennessee Walking horses/Saddlebreds (22 horses, 14%), Thoroughbreds (18 horses, 12%), Warmbloods (16 horses, 11%), Arabians (12 horses, 8%), draft horses (8 horses, 6%), ponies (7 animals 5%) and mixed, and other breeds (8 horses, 6%), reflecting the hospital populations.

### 3.2 Clinical data

The most common clinical signs reported were tachycardia (82 horses, 55%), tachypnea (67 horses, 46%), prolonged capillary refill time (42 horses, 28%), hypothermia (25 horses, 17%), and pyrexia (18 horses, 7%). Dehydration was recorded in 93/131 horses (71%) with a median estimation of 6% (4‐12). Nasogastric reflux was present in 33/127 horses (26%) with a median volume of 7 L (2‐19). Rectal palpation was performed in 134/151 horses (89%) and in 110 horses (82%) abnormal findings were described. The most commonly reported abnormal findings were distention of small intestine (48 horses, 43%), large colon impaction (26 horses, 17%), and large colon displacement (37 horses, 25%). A complete blood count was available in 134 horses (89%) and a serum chemistry profile was available in 107 horses (71%). The most commonly reported abnormalities were hyperglycemia (62/109 horses, 57%), neutrophilia (79/134, 59%), hypokalemia (55/107, 51%), hyperlactatemia (27/55, 49%), hyponatremia (51/107, 48%), low base excess (21/48, 44%), low ionized calcium (20/52, 38%), hyperfibrinogenemia (28/91, 31%), hypoalbuminemia (25/84, 30%), increased creatinine (32/131, 24%), leukocytosis (27/134, 20%), decreased bicarbonate (20/103, 19%), leukopenia (20/134, 15%), and neutropenia (19/134, 14%).

The final diagnosis involved the gastrointestinal system in 144 horses (97), the respiratory system in 3 horses (2%), and the reproductive system and the neurologic system in 1 case each (<1% each). Forty‐five horses with a gastrointestinal disease (31%) had an explorative celiotomy and, based on surgery (27 horses) or necropsy reports (23 horses), 50 (33%) had an ischemic lesion. One hundred six horses (70%) were discharged alive. No horses died spontaneously, but 45 horses were euthanized as the attending specialist advised the prognosis was poor to hopeless. Thirty cases were euthanized either at admission or during the first evening (4 of which had ruptured bowel at admission and 4 additional cases were euthanized during exploratory celiotomy). Of the 6 nonsurvivors that lived 6 or more days, only 4 were sampled on every occasion. Of the nonsurvivors, 32/45 (71%) had SIRS, whereas 44/106 (41%) survivors had SIRS. The median SIRS score in nonsurvivors was significantly higher than in survivors (2 [0‐4] versus 1 [0‐4], *P* < .0001). The survival rates for each of the SIRS scores and groups are shown in Table 1.

**Table 1:** Distribution of survivors and nonsurvivors between SIRS scores 0‐4 and SIRS groups, SIRS 0/SIRS 1, SIRS 2, and SIRS 3/SIRS 4

	Survivors (106)			Nonsurvivors (45)		
SIRS 0	26	62	62 (59%)	1	13	13 (29%)
SIRS 1	36			12		
SIRS 2	30	30	44 (41%)	14	14	32 (71%)
SIRS 3	10	14		11	18	
SIRS 4	4			7		

## 4 SURVIVAL

### 4.1 Adrenocorticotropic hormone

**Figure 1:**
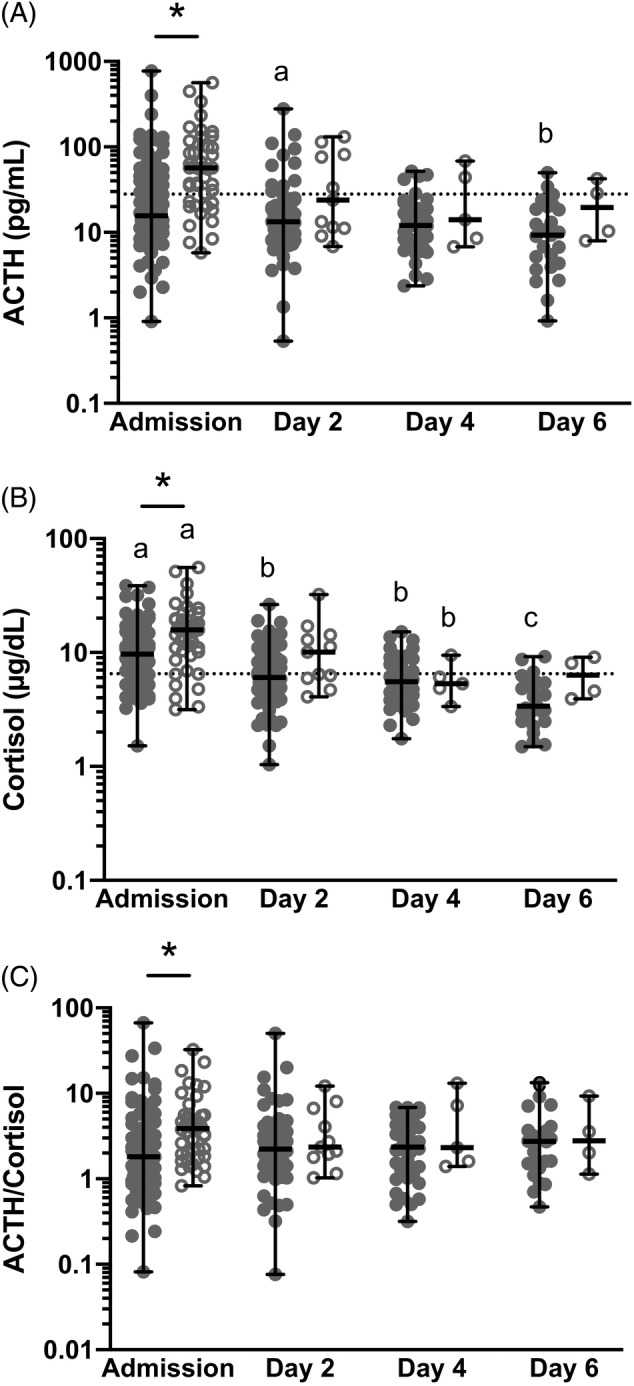
A‐C, Plasma adrenocorticotropic hormone (ACTH, A), serum cortisol (B), and ACTH/cortisol ratio (C) at admission, day 2, day 4, and day 6 of hospitalization in survivors (solid circles) versus nonsurvivors (hollow circles). *Difference between survivors and nonsurvivors for ACTH (*P* < .0009), cortisol (*P* = .012), and ACTH/cortisol ratio (*P* = .0001). The reference range is marked by a dotted horizontal line. A different letter indicates a significant difference between days (*P* < .05)

There was a significant effect of time and survival status on ACTH concentration, with higher ACTH concentrations at admission overall (*P* = .006) and in nonsurvivors (*P* = .009). In survivors, ACTH concentrations on day 2 were significantly higher than on day 6 (*P* = .021). At admission, ACTH concentrations were significantly higher in nonsurvivors than in survivors (*P* = .009, Figure 1A and Table S1).

Of the surviving horses, 86% (71/83) had an ACTH concentration within the reference range at admission, whereas 49% of horses (32/65) with ACTH outside the reference range survived. The proportion of horses with ACTH concentrations below reference range for each day of hospitalization is shown in Table 2. Of the surviving horses, 92% (95/103) had a normal ACTH concentration before hospital discharge. When the ACTH concentration was outside the reference range at admission the OR and 95% CI for nonsurvival was 6.10 (2.73‐13.68), *P* < .0001. The ROC curve showed that a plasma ACTH concentration > 30.5 pg/mL (the algorithm's suggested optimal cutoff) was a fair diagnostic test to predict survival with an area under the ROC of 0.75, with sensitivity of 71.1% (95% CI = 55.7‐83.6) and specificity of 71.8% (62.1‐80.3) and a likelihood ratio of 2.53 for nonsurvival (*P <* .0001). On day 2, 91.2% of horses (62/68) with ACTH within reference range survived, while 68.6% of horses (11/16) with ACTH outside reference range survived. For ACTH concentration outside reference range on day 2, the OR for nonsurvival was 4.70 (1.26‐15.54), *P* = .016.

**Table 2:** Proportion of horses with ACTH below the laboratories reference range of <28 pg/mL on each day of hospitalization

Proportion of horses with ACTH <28 pg/mL	Day 0	Day 2	Day 4	Day 6
All horses	83/148 = 56.1%	68/84 = 80.9%	43/50 = 86%	25/29 = 86.2%
Surviving horses	71/103 = 68.9%	62/73 = 84.9%	40/45 = 88.9%	23/25 = 92%
Nonsurviving horses	12/45 = 26.7%	6/11 = 54.5%	3/5 = 60%	2/4 = 50%

### 4.2 Cortisol

There was a significant effect of time and survival status on cortisol concentration with higher cortisol at admission overall (*P* < .0001) and in nonsurvivors (*P* = .011). In survivors, cortisol concentrations were significantly higher at admission than on days 2, 4, and 6 (*P* < .0001, for all comparisons), on day 2 compared to day 6 (*P* < .0001), and on day 4 compared to day 6 (*P* = .0006). In nonsurvivors, cortisol concentrations at admission were significantly higher than on day 4 (*P* = .0061). At admission, cortisol concentrations were significantly higher in nonsurvivors than in survivors (*P* = .011, Figure 1B and Table S1).

The ROC curve showed that a serum cortisol concentration >11.7 μg/dL was a poor diagnostic test to predict survival with an area under the ROC of 0.68, with sensitivity of 68.9% (95% CI = 53.4‐81.8), specificity of 67.3% (57.4‐76.2; *P* = .0004), and a likelihood ratio of 2.11 for nonsurvival.

### 4.3 ACTH/cortisol ratio

There was no significant effect of time on ACTH/cortisol (*P* = .6801); however, there was a significant effect of survival (*P =* .0001). At admission, ACTH/cortisol ratios were significantly higher in nonsurvivors than in survivors (*P* = .0001, Figure 1C and Table S1).

The ROC curve showed that an ACTH/cortisol ratio concentration > 2.6 was a poor diagnostic test to predict survival with an area under the ROC of 0.696, with sensitivity of 64.4 (95% CI = 48.8‐78.1), specificity of 67.3% 67.3 (57.3‐76.3) and a likelihood ratio of 1.97 for nonsurvival (*P =* .0002).

## 5 SIRS SCORES AND GROUPS

### 5.1 Adrenocorticotropic hormone

#### 5.1.1 SIRS versus no SIRS

**Figure 2:**
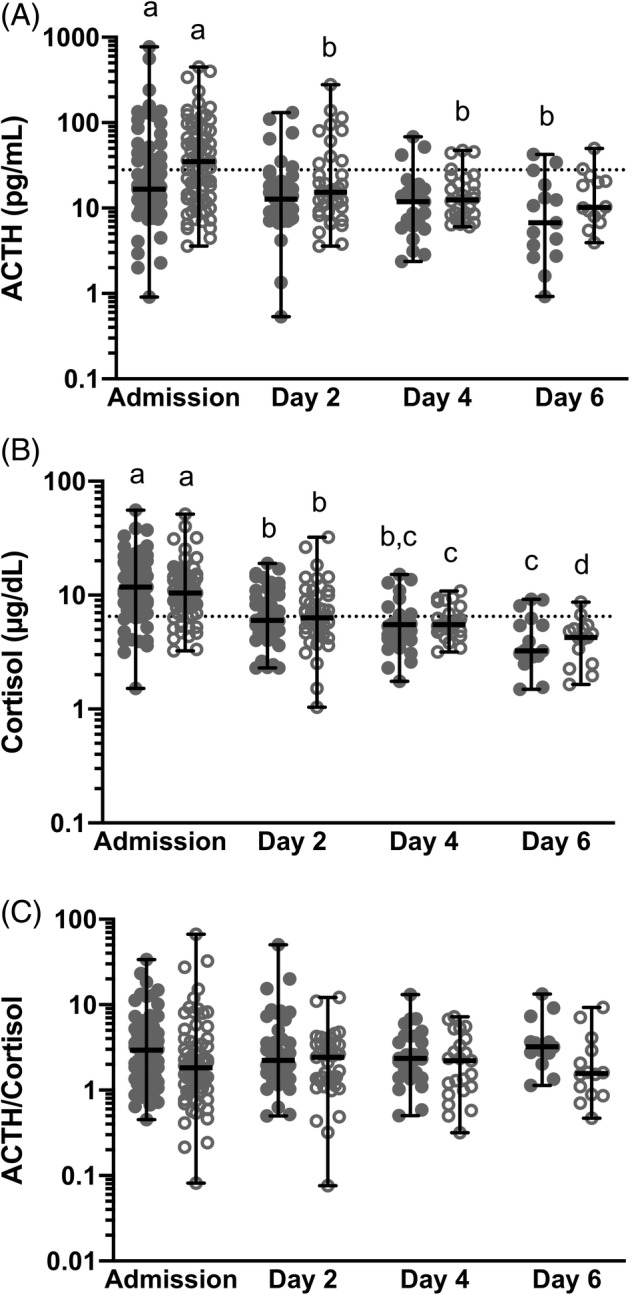
A‐C, Plasma adrenocorticotropic hormone (ACTH, A), serum cortisol (B), and ACTH/cortisol ratio (C) at admission, day 2, day 4, and day 6 of hospitalization for horses with systemic inflammatory response syndrome (SIRS, hollow circles) verses without SIRS (solid circles). The presence of SIRS is defined as having ≥2 SIRS criteria which are defined as: heart rate >52 bpm, respiratory rate >20 bpm, temperature below or above 37.0–38.5°C, and WBC below or above 5.0–12.5 × 10^9^/L (or the presence of band neutrophils). The reference range is marked by a dotted horizontal line. A different letter indicates a significant difference between days (*P* < .05)

There was a significant effect of time on ACTH concentrations with higher ACTH concentrations at admission (*P* = .008); however, there was no effect of having SIRS (*P* = .57). In horses with no SIRS, ACTH concentrations at admission were significantly higher than on day 6 (*P* = .006). In horses with SIRS, ACTH concentrations at admission were significantly higher than on days 2 and 4 (*P* = .048 and .0032, respectively, Figure 2A and Table S2).

#### 5.1.2 SIRS scores

There was a significant effect of time on ACTH concentrations with higher ACTH concentrations at admission (*P* = .036); however, there was no effect of SIRS score (*P* = 0.94). In horses with a SIRS score of 1, ACTH concentrations were significantly higher at admission than on day 6 (*P* = .021) and on day 2 compared to days 4 and 6 (*P* = 0.024 and 0.029, respectively, Figure S1A and Table S3).

#### 5.1.3 SIRS groups

There was a significant effect of time on ACTH concentrations with higher ACTH concentrations at admission (*P* = .013); however, there was no effect of SIRS group (*P* = .80). In horses without SIRS (SIRS 1/SIRS 0), ACTH concentrations were significantly higher at admission than on day 6 (*P* = .005) and on day 2 compared to day 6 (*P* = .039). In horses with SIRS 3/SIRS 4, ACTH concentrations at admission were significantly higher than on day 4 (*P* = .013, Figure S2A and Table S4).

### 5.2 Cortisol

#### 5.2.1 SIRS versus no SIRS

There was a significant effect of time on cortisol concentrations with higher cortisol concentrations at admission (*P* < .0001); however, there was no effect of having SIRS (*P* = .41). In horses with no SIRS, cortisol concentrations were significantly higher at admission than on days 2, 4, and 6 (*P* < .0001 for all comparisons) and on day 2 compared to day 6 (*P* = .0356). In horses with SIRS, cortisol concentrations were significantly higher at admission than on days 2, 4, and 6 (*P* < .0001 for all comparisons); on day 2 compared to days 4 and 6 (*P* = .0237 and <.0001, respectively) and on day 4 compared to day 6 (*P* = .0382, Figure 2B and Table S2).

#### 5.2.2 SIRS scores

There was a significant effect of time on cortisol concentrations with higher cortisol concentrations at admission (*P* < .0001); however, there was no effect of SIRS score (*P* = .85). In horses with a SIRS score of 0, cortisol concentrations at admission were significantly higher than on day 2, 4, and 6 (*P* = .005, .027, and .041, respectively). In horses with a SIRS score of 1, cortisol concentrations were significantly higher at admission than on days 2, 4, and 6 (*P* = .004, .0004, and .0002, respectively), on day 2 than day 6 (*P* = .0005), and on day 4 than day 6 (*P* = .0075). In horses with a SIRS score of 2, cortisol concentrations at admission were significantly higher than on days 2, 4, and 6 (*P* = .005, .0009, and .024, respectively). In horses with a SIRS score of 3, cortisol concentrations at admission were significantly higher than on days 2 and 4 (*P* = .002 and .0002, respectively, Figure S1B and Table S3).

#### 5.2.3 SIRS groups

There was a significant effect of time on cortisol concentrations with higher cortisol concentrations at admission (*P* < .0001); however, there was no effect of SIRS group (*P* = .68). In horses without SIRS (SIRS 1/SIRS 0), cortisol concentrations were significantly higher at admission than on days 2, 4, and 6 (*P* < .0001 for all comparisons); on day 2 compared to days 4 and 6 (*P* = .019 and <.0001) and on day 4 compared to day 6 (*P* = .029). In horses with SIRS 2, cortisol concentrations at admission were significantly higher than on days 2, 4, and 6 (*P* = .004, .0007, and .022, respectively). In horses with SIRS 3/SIRS 4, cortisol concentrations at admission were significantly higher than on days 2, 4, and 6 (*P* < .0001, .0002, and .009, respectively, Figure S2B and Table S4).

### 5.3 ACTH/cortisol ratio

#### 5.3.1 SIRS versus no SIRS

There was no significant effect of time or having SIRS on ACTH/cortisol ratio (*P* = .37 and .22, respectively, Figure 2C and Table S2).

#### 5.3.2 SIRS scores

There was no significant effect of time or SIRS score on ACTH/cortisol ratio (*P* = .51 and .46, respectively, Figure S1C and Table S3).

#### 5.3.3 SIRS group

There was no significant effect of time or SIRS group on ACTH/cortisol ratio (*P* = .41 and .16, respectively, Figure S2C and Table S4).

## 6 ISCHEMIC GASTROINTESTINAL LESIONS

Of the 50 horses with ischemic lesions, only 36% (18/50) survived, while 87.1% (88/101) with nonischemic lesions survived. Horses with an ischemic lesion were 12 times more likely not to survive (OR: 12.03 [5.36‐25.54], *P* < .0001). Median SIRS score in horses with an ischemic lesion was significantly higher than in horses with no ischemic lesion (2 [0‐4] versus 1 [0‐4], *P* = .025).

### 6.1 Adrenocorticotropic hormone

**Figure 3:**
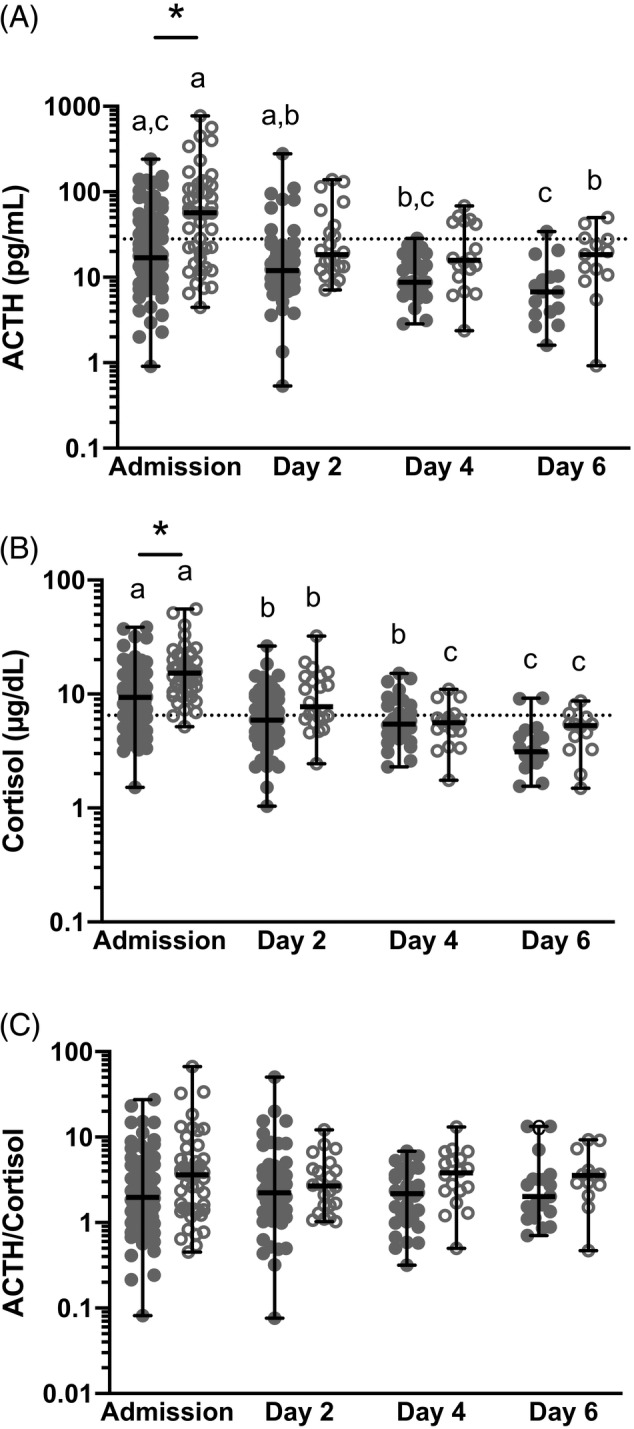
A‐C, Plasma adrenocorticotropic hormone (ACTH, A), serum cortisol (B), and ACTH/cortisol ratio (C) at admission, day 2, day 4, and day 6 of hospitalization in horses with ischemic gastrointestinal lesions (open circles) versus horses without ischemic lesions (full circles). *Difference between ischemic and nonischemic for ACTH (*P* = .0061) and cortisol (*P* = .0009). The reference range is marked by a dotted horizontal line. A different letter indicates a significant difference between days (*P* < .05)

There was a significant effect of time and ischemia on ACTH concentrations with higher ACTH concentrations overall at admission (*P* = .0008) and in horses with an ischemic lesion (*P* = .008). In horses with no ischemic lesion, ACTH concentrations were significantly higher at admission than on day 4 (*P* = .0003) and on day 2 compared to day 6 (*P* = .038). In horses with an ischemic lesion, ACTH concentrations were significantly higher at admission than on day 6 (*P* = .013). At admission, horses with an ischemic lesion had significantly higher ACTH concentrations than horses without (*P* = .006, Figure 3A and Table S5). The ROC curve showed that an ACTH concentration > 28.5 pg/mL was a poor diagnostic test to predict ischemia with an area under the ROC of 0.73, with sensitivity of 66.7% (52.1‐79.2), specificity of 69.1% (58.9‐78.1), and a likelihood ratio of 2.16 for the presence of an ischemic lesion (*P* < .0001).

### 6.2 Cortisol

There was a significant effect of time and ischemia on cortisol concentrations with higher cortisol concentrations at admission overall (*P* < .0001) and in horses with an ischemic lesion (*P* = .0007). In horses with no ischemic lesion, cortisol concentrations were significantly higher at admission than on days 2, 4, and 6 (*P* < .0001, .0001, and .001, respectively), on day 2 compared to day 6 (*P* = .002) and on day 4 to day 6 (*P* = .019). In horses with an ischemic lesion, cortisol concentrations were significantly higher at admission than on days 2, 4, and 6 (*P* = .0006, .0001 and .0001, respectively), on day 2 compared to days 4 and 6 (*P* = .0011 and .0002, respectively). At admission, horses with an ischemic lesion had significantly higher cortisol concentrations than horses without (*P* = .0009, Figure 3B and Table S5).

The ROC curve showed that a cortisol concentration > 11.6 μg/dL was a poor diagnostic test to predict ischemia with an area under the ROC of 0.73, with sensitivity of 70.6% (56.2‐82.5), specificity of 69.4% (59.3‐78.3) and a likelihood ratio of 2.31 for the presence of an ischemic lesion (*P* < .0001).

### 6.3 ACTH/cortisol ratio

There was no significant effect of time or the presence of an ischemic lesion on ACTH/cortisol ratio (*P* = .23 and .21, respectively, Figure 3C and Table S5). The ROC curve showed that an ACTH/cortisol ratio > 2.4 was a poor diagnostic test to predict ischemia with an area under the ROC of 0.63, with sensitivity of 62.8% (48.1‐75.9), specificity of 64.2% (53.7‐73.8) and a likelihood ratio of 1.75 for the presence of an ischemic lesion (*P* = .0071).

## 7 DISCUSSION

Cortisol and ACTH concentrations were higher in nonsurviving horses and those with an ischemic gastrointestinal lesion. The ACTH/cortisol ratio was higher in nonsurviving horses. When ACTH concentrations were within reference range on admission or day 2, horses had 6.1 or 4.2 times higher likelihood of survival. The ideal ACTH cutoff value for predicting survival was > 30.5 pg/mL, with a sensitivity and specificity of only 71% and 72%. Cortisol was a poorer predictor of survival, where a cutoff value of > 11.7 μg/dL had sensitivity and specificity of 69% and 67%. Therefore, ACTH and cortisol concentrations should not be used as sole predictors of survival. Although the pituitary gland has a high capacity to increase ACTH production, in surviving animals this response was rapidly normalized, with 92% of horses having ACTH within reference interval before hospital discharge. Overall, both ACTH and cortisol decreased over time and the effect was more marked in nonsurvivors. Although the SIRS score was higher in nonsurvivors and horses with ischemia, there was no effect of SIRS status, SIRS score, or SIRS groups on ACTH, cortisol, or ACTH/cortisol ratio.

Several scoring systems have been compared to categorize the severity of illness and likelihood of survival in septic foals, allowing comparison between research studies.^22^, ^23^ There is no accepted grading scheme to categorize the severity of illness in adult horses, although a scoring system for multiple organ dysfunction in equine surgical patients was recently developed.^24^ An equine SIRS score validated using a primary referral practice had a SIRS rate of 31.3% of all emergency admissions, 24% of colic cases, and 67% of horses with a strangulating gastrointestinal lesion.^18^ In our study, SIRS was diagnosed in 50% of horses, 58% of colics with ischemic lesions, and 71% of nonsurvivors. The diagnostic utility of this SIRS score was improved with the addition of blood lactate and mucous membrane color.^18^ A colic severity score utilizing heart rate, peritoneal fluid total protein, and blood lactate had a 93% accuracy at predicting survival.^25^ Unfortunately, blood lactate concentration was available for less than half of our horses, and peritoneal fluid total protein for 60% of cases.

In critically ill humans, the APACHE‐II score assesses illness severity and risk of death.^26^ However, day 2 cortisol concentration was a better discriminator of ICU patient outcome with a predictive ability of 81%, while the APACHE‐II score had a predictive ability of only 70%.^27^, ^28^ Although ACTH, cortisol, and ACTH/cortisol ratios were higher in our nonsurvivors, no single factor should be used to predict survival of individual cases. Experienced equine clinicians are also better predictors of survival than scoring systems.^29^ Although many studies have investigated admission clinicopathologic findings to assess likelihood of survival, changes over time, in horses surviving the initial admission period, might be more important. We attempted to assess cortisol and ACTH concentrations over 6 days of hospitalization, but many nonseverely ill horses were discharged before day 6, and the majority of our nonsurvivors were euthanized within the initial 24 hours, with only 11 nonsurvivors available for testing on day 2, which was underpowered to show a difference between survivors and nonsurvivors.

Equine emergency patients experience stress from fear, pain, dehydration, inappetence, and consequences of systemic inflammation such as fever, hypotension, or hypoxemia. Factors affecting CRH secretion in a variety of species include cytokines, lipopolysaccharide, brain‐derived neurotropic factor, neurosteroids, glutamate, γ‐aminobutyric acid, nitric oxide, and glucocorticoids (through negative feedback).^30^ Lipopolysaccharide was detected in 29% of horses presenting for colic, and is more commonly detected in horses with ischemic gastrointestinal lesions.^31^ Interleukins‐1β, ‐6, and ‐8 mRNA expression have been shown to be upregulated in surgical colic patients.^32^ Neurosteroids have been measured in critically ill foals, but effects of inflammatory mediators on the HPA axis of sick horses has not been investigated. Arginine vasopressin and CRH stimulate ACTH release from corticotropes in the pituitary *pars distalis*, which is moderated by cytokines, lipopolysaccharide, nitric oxide, and glucocorticoids during illness.^30^ We did not assess CRH or vasopressin concentrations but the prevalence of ACTH hyposecretion or hypothalamic dysregulation in relation to HPA axis dysfunction appears a rare cause of CIRCI compared to adrenocortical failure in humans and septic foals.^4^, ^5^

Studies assessing ACTH concentrations in sick adult horses are sparse, with mean ACTH concentration 2.4 times higher in 43 horses with colic compared to normal controls, with highest values in horses with strangulated intestine.^10^ Similarly, we found ACTH was significantly higher (3.4 times) in horses with ischemic gastrointestinal lesions than those without. Median ACTH in nonsurviving horses was 3.6 times higher than normal horses (from our institution measured with the same assay^20^). However, individual variation in sick horses was extreme, with the highest ACTH occurring in a surviving horse, which was 49 times higher than normal horses. In comparison to adult horses, foals have lower ACTH concentrations and ACTH is higher in septic compared to healthy foals and higher in nonsurvivors than survivors.^3^, ^4^, ^33^, ^34^

The adrenal cortex *zona fasciculata* secretes glucocorticoids (cortisol), which decrease CRH, vasopressin, and ACTH secretion by negative feedback. Cortisol increases gluconeogenesis, decreases glucose utilization, increases serum glucose, and inhibits the effects of insulin. Median cortisol was increased in nonsurvivors and those with ischemic gastrointestinal lesions. These findings were supported by previous studies in horses with colic.^10^, ^35^ Mair showed that cortisol > 7.25 μg/dL was more common in horses with colic than healthy controls; that cortisol was higher in horses with colic with a heart rate > 45 beats per minute, and that a higher cortisol was associated with more severe signs of colic.^11^ This study was unable to detect a direct association between high cortisol and non‐survival.^11^

We were expecting disease severity, as defined by SIRS status/score/group to significantly affect ACTH and cortisol concentrations, but this was not identified. The original SIRS definitions were intended to identify human patients with severe disease.^36^ Other scoring systems such as the APACHE‐II are designed to grade disease severity.^37^ Although SIRS status, scores, or groups are useful to differentiate horses with and without systemic illness,^14^, ^18^ horses without systemic illness (wounds, fractures, and ocular emergencies), were not included in our study. Although illness severity was higher, the spread of severities in our study population was less than the primary referral equine SIRS validation study.^18^ Alternatively, the reason that we failed to find associations with SIRS status/score/group in comparison to hormone concentrations might be caused by CIRCI. A subset of critically ill horses with inappropriately low endogenous cortisol would cancel out appropriately high cortisol concentrations during statistical analysis. In a separate smaller study in which ACTH stimulation tests were performed on systemically ill horses, we determined that cortisol <4.51 μg/dL was inappropriately low^38^ (mean − 1 standard deviation obtained from healthy horses).^34^, ^39^ With this cutoff in the current study, 11/149 (7.4%) of horses would have inappropriately low cortisol at admission, 19/90 (21.1%) on day 2, 16/55 (29.1%) on day 4, and 19/31 (61.3%) on day 6. Regardless of illness severity, sick hospitalized horses should not have a cortisol concentration less than that of stall acclimatized, university‐owned teaching horses.

Basal cortisol is lower in normal neonatal foals than adult horses, but adult horses have greater adrenal reserve with increased cortisol release after exogenous ACTH stimulation.^33^, ^34^ Our sick adult horses had higher cortisol concentrations than those observed in septic neonatal foals.^3^, ^4^

When pituitary reserve and ability to release ACTH exceeds the corresponding capacity for adrenal production and release of cortisol, a high ACTH/cortisol ratio results indicating ACTH‐cortisol imbalance.^12^, ^14^ Adrenal gland dysfunction or RAI/CIRCI is identified when cortisol release is inadequate for illness severity. Although ACTH/cortisol ratio is not a criteria for the diagnosis of CIRCI in humans, and is not as accurate as an ACTH stimulation test,^4^, ^5^ it is easily performed, inexpensive and has provided useful information in critically ill foals.^3^, ^8^, ^9^ We found a higher ACTH/cortisol ratio in nonsurvivors, while others have found higher ACTH/cortisol ratios in septic compared to sick‐nonseptic or normal foals.^3^, ^12^ The value is much higher in foals because healthy and sick foals have higher ACTH and lower cortisol than adult horses.^33^, ^34^ In a small study of 10 horses with SIRS, nonsurvivors had an increased (but not statistically significant) ACTH/cortisol ratio and a decrease in glucocorticoid receptor binding affinity, which suggests tissue resistance to glucocorticoids.^14^

An appropriate cortisol concentration in response to illness of varying severities has not been defined for adult horses. In humans and foals, a low baseline cortisol concentration or an inadequate response to a high‐ or low‐dose ACTH stimulation test have been advocated as means to diagnose CIRCI.^5^, ^8^, ^9^ However, in human medicine, because of the low sensitivity and specificity of the results of endocrine testing to diagnose CIRCI, current recommendations are to administer low‐dose corticosteroids to treat adult patients with septic shock that are nonresponsive to fluid therapy or moderate to high doses of vasopressors rather than waiting on endogenous or ACTH‐stimulated cortisol test results.^5^

This study could have been improved by a larger sample size and serial assessments of blood pressure and lactate and adrenal and pituitary histopathology on all nonsurvivors. Overall, higher concentrations of ACTH, cortisol, and ACTH/cortisol ratio were associated with nonsurvival and higher ACTH and cortisol with the presence of an ischemic lesion. There was a subset of horses with inappropriately low cortisol suggesting the presence of RAI/CIRCI. Inflammation, shock, ischemia/reperfusion injury, and SIRS are common syndromes requiring multimodal medical treatment in horses with colic. Inappropriately low basal cortisol concentrations, especially with a high ACTH/cortisol ratio could indicate pituitary‐adrenal dysregulation and possible RAI/CIRCI.^3^, ^14^ This requires further investigation in severely ill horses in conjunction with assessment of ACTH‐stimulated cortisol concentrations before treatment is considered.

## CONFLICT OF INTEREST DECLARATION

Authors declare no conflict of interest.

## OFF‐LABEL ANTIMICROBIAL DECLARATION

Authors declare no off‐label use of antimicrobials.

## INSTITUTIONAL ANIMAL CARE AND USE COMMITTEE (IACUC) OR OTHER APPROVAL DECLARATION

All aspects of the study were approved by the Auburn and Colorado State Universities Institutional Laboratory Animal Care and Use Committees (2009‐1564, 09‐079A‐01) and the College of Veterinary Medicine Clinical Research Review Committee. Signed owner's consent was obtained for all procedures.

## HUMAN ETHICS APPROVAL DECLARATION

Authors declare human ethics approval was not needed for this study.

## Supporting information


**FIGURE S1**A‐C, Plasma ACTH (A), serum cortisol (B), and ACTH/cortisol ratio (C) at admission, day 2, day 4, and day 6 of hospitalization in horses with SIRS scores 0‐4. The SIRS score is based on the number of abnormal SIRS criteria based on heart rate > 52 bpm, respiratory rate > 20 bpm, temperature below or above 37.0‐38.5°C, and WBC below or above 5.0‐12.5 × 10^9^/L. From right to left on each day SIRS score 0 (SIRS0), SIRS1, SIRS2, SIRS3, and SIRS4. The reference range is marked by a dotted horizontal line. A different letter indicates a significant difference between days (*P* < .05)

**FIGURE S2**A‐C, Plasma ACTH (A), serum cortisol (B), and ACTH/cortisol ratio (c) at admission, day 2, day 4, and day 6 of hospitalization in horses with no SIRS (0‐1 abnormal criteria); SIRS2: 2 abnormal SIRS criteria; SIRS 3/SIRS 4:3 or 4 abnormal SIRS criteria. The SIRS criteria are defined as: heart rate > 52 bpm, respiratory rate > 20 bpm, temperature below or above 37.0–38.5°C, and WBC below or above 5.0‐12.5 × 10^9^/L. From right to left on each day SIRS0, SIRS1/2, and SIRS 3/SIRS 4. The reference range is marked by a dotted horizontal line. A different letter indicates a significant difference between days (*P* < .05)

**Table S1.** Changes in the median [range] plasma ACTH and serum cortisol concentrations and the ACTH/cortisol ratio for survivors and nonsurvivors over 6 days of hospitalization
**Table S2.** Changes in the median [range] plasma ACTH and serum cortisol concentrations and the ACTH/cortisol for horses with and without SIRS over 6 days of hospitalization
**Table S3.** Changes in the median [range] plasma ACTH and serum cortisol concentrations and the ACTH/cortisol ratio for SIRS score categories (0‐4) over 6 days of hospitalization
**Table S4**. Changes in the median [range] plasma ACTH and serum cortisol concentrations and the ACTH/cortisol ratio for horses with no SIRS (0‐1 abnormal criteria); SIRS2: 2 abnormal SIRS criteria; SIRS 3/SIRS 4:3 or 4 abnormal SIRS criteria over 6 days of hospitalization
**Table S5.** Changes in the median [range] plasma ACTH and serum cortisol concentrations and the ACTH/cortisol for horses with and without an ischemic gastrointestinal lesion over 6 days of hospitalization
