# Supplementary figures and images for: Cortisol and adrenocorticotropic hormone concentrations in horses with systemic inflammatory response syndrome
Source: J Vet Intern Med. 2019 Sep 12;33(5):2257–66. doi: 10.1111/jvim.15620 (PMC6766528; doi:10.1111/jvim.15620)

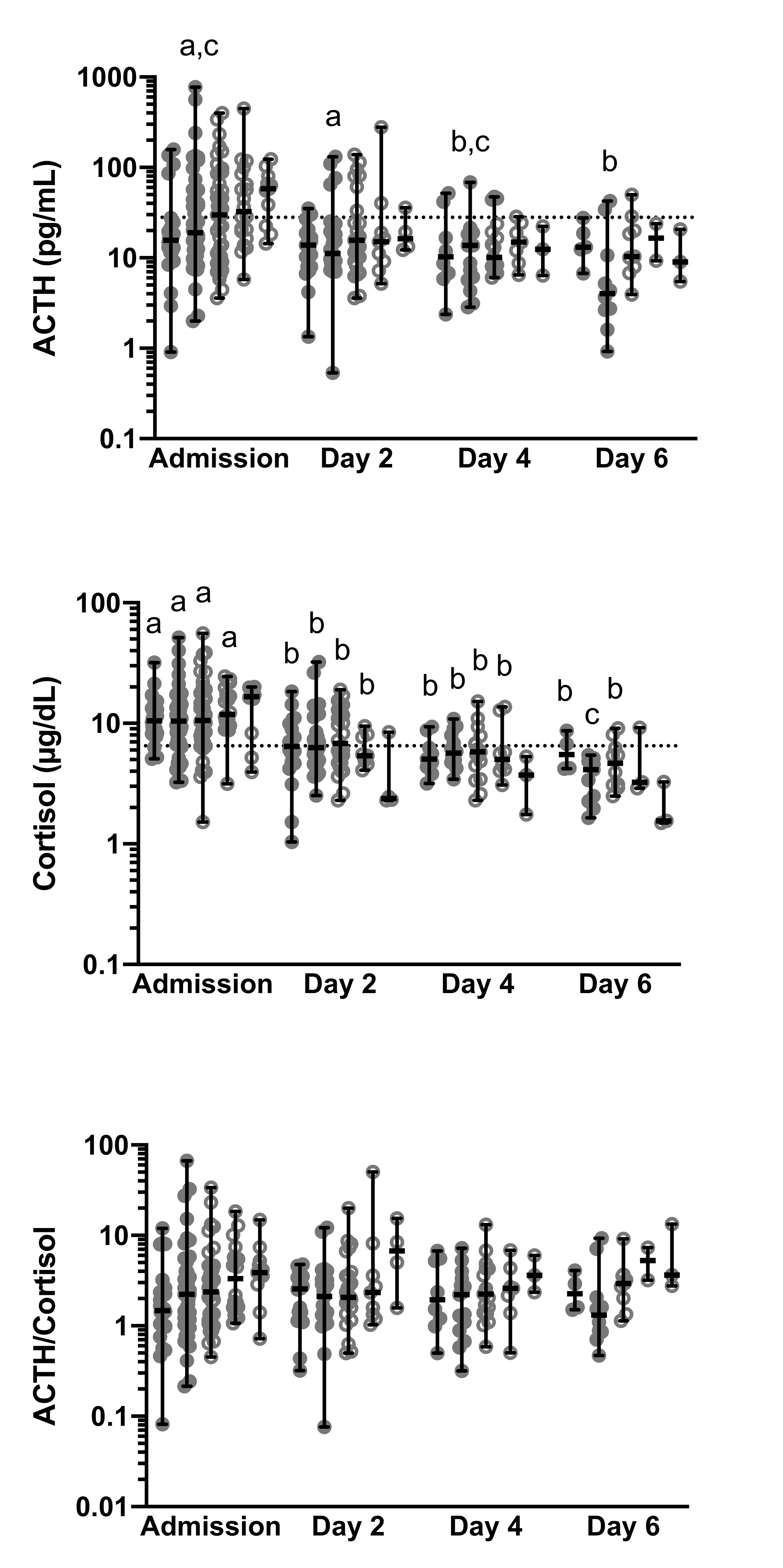

Supplement: Supplementary file 1 — FIGURE S1A‐C, Plasma ACTH (A), serum cortisol (B), and ACTH/cortisol ratio (C) at admission, day 2, day 4, and day 6 of hospitalization in horses with SIRS scores 0‐4. The SIRS score is based on the number of abnormal SIRS criteria based on heart rate > 52 bpm, respiratory rate > 20 bpm, temperature below or above 37.0‐38.5°C, and WBC below or above 5.0‐12.5 × 109/L. From right to left on each day SIRS score 0 (SIRS0), SIRS1, SIRS2, SIRS3, and SIRS4. The reference range is marked by a dotted horizontal line. A different letter indicates a significant difference between days (P < .05) [file JVIM-33-2257-s001.jpg]

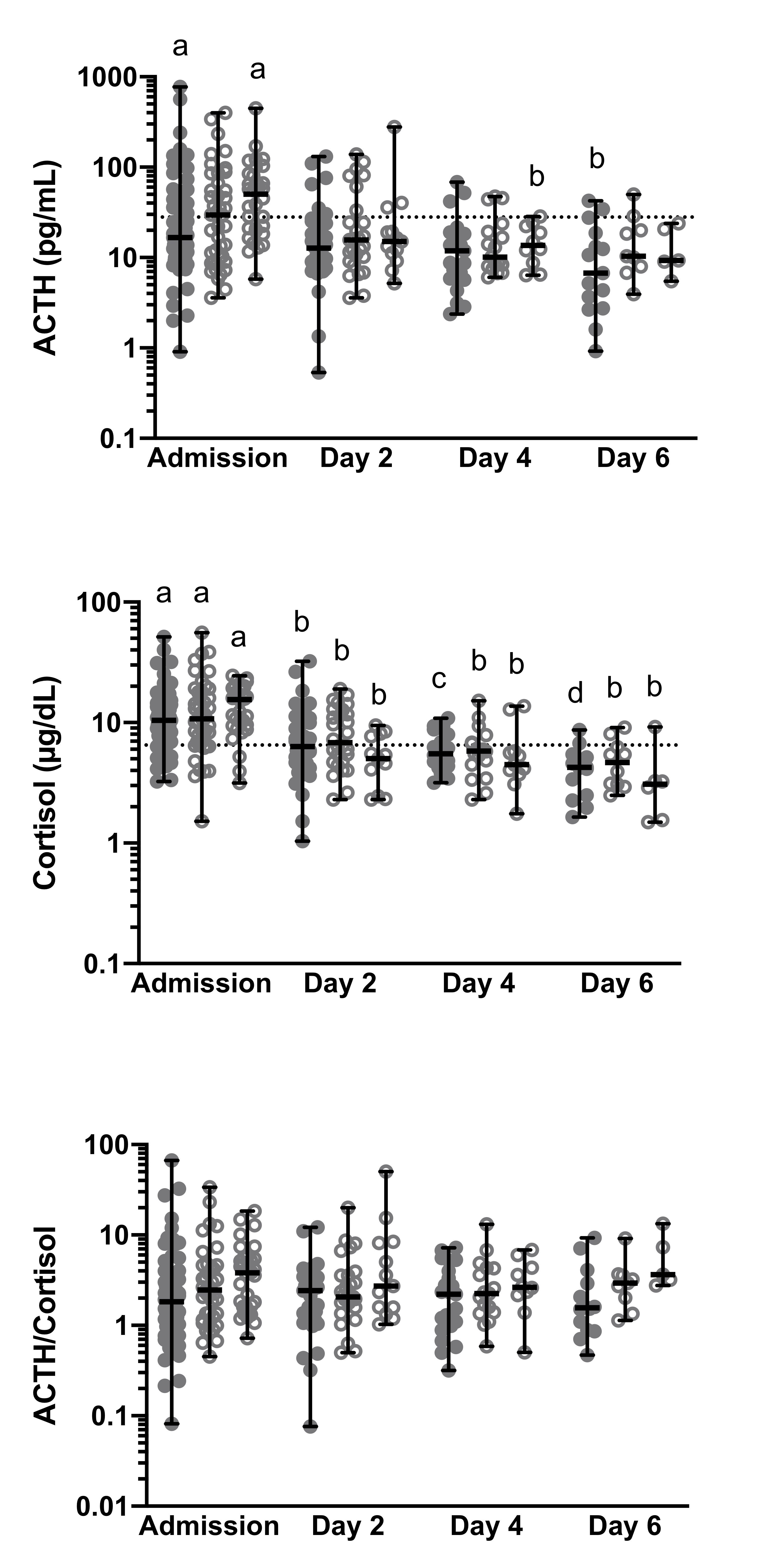

Supplement: Supplementary file 2 — FIGURE S2A‐C, Plasma ACTH (A), serum cortisol (B), and ACTH/cortisol ratio (c) at admission, day 2, day 4, and day 6 of hospitalization in horses with no SIRS (0‐1 abnormal criteria); SIRS2: 2 abnormal SIRS criteria; SIRS 3/SIRS 4:3 or 4 abnormal SIRS criteria. The SIRS criteria are defined as: heart rate > 52 bpm, respiratory rate > 20 bpm, temperature below or above 37.0–38.5°C, and WBC below or above 5.0‐12.5 × 109/L. From right to left on each day SIRS0, SIRS1/2, and SIRS 3/SIRS 4. The reference range is marked by a dotted horizontal line. A different letter indicates a significant difference between days (P < .05) [file JVIM-33-2257-s002.jpg]
