# Supplementary material for: Cortisol and adrenocorticotropic hormone concentrations in horses with systemic inflammatory response syndrome
Source: J Vet Intern Med. 2019 Sep 12;33(5):2257–66. doi: 10.1111/jvim.15620 (PMC6766528; doi:10.1111/jvim.15620)
Supplement: Supplementary file 3 — Table S1. Changes in the median [range] plasma ACTH and serum cortisol concentrations and the ACTH/cortisol ratio for survivors and nonsurvivors over 6 days of hospitalization Table S2. Changes in the median [range] plasma ACTH and serum cortisol concentrations and the ACTH/cortisol for horses with and without SIRS over 6 days of hospitalization Table S3. Changes in the median [range] plasma ACTH and serum cortisol concentrations and the ACTH/cortisol ratio for SIRS score categories (0‐4) over 6 days of hospitalization Table S4. Changes in the median [range] plasma ACTH and serum cortisol concentrations and the ACTH/cortisol ratio for horses with no SIRS (0‐1 abnormal criteria); SIRS2: 2 abnormal SIRS criteria; SIRS 3/SIRS 4:3 or 4 abnormal SIRS criteria over 6 days of hospitalization Table S5. Changes in the median [range] plasma ACTH and serum cortisol concentrations and the ACTH/cortisol for horses with and without an ischemic gastrointestinal lesion over 6 days of hospitalization [file JVIM-33-2257-s003.pdf]

Supplementary data Table 1. Changes in the median [range] plasma ACTH and serum cortisol concentrations and the ACTH/cortisol ratio for survivors and non-survivors over 6 days of hospitalization.

	Admission	Day 2	Day 4	Day 6
ACTH (pg/mL)				
Total population	21.6[a] [0.9 – 775.1] (n = 148)	13.6 [0.5 – 278.0] (n = 84)	12.2 [2.4 – 68.5] (n = 50)	10.1[b] [0.9 – 49.9] (n = 29)
Survivors	15.7* [0.9 – 775.1] (n = 103)	13.3[a] [0.5 – 278.0] (n = 73)	12.0 [2.4 – 51.9] (n = 45)	9.3[b] [0.9 – 49.9] (n = 25)
Non-survivors	56.7* [5.8 – 563.8] (n = 45)	23.9 [6.8 – 131.6] (n = 11)	14.1 [6.8 – 68.5] (n = 5)	19.5 [7.9 – 42.5] (n = 4)
Cortisol (µg/dL)				
Total population	11.0[a] [1.5 – 55.85] (n = 149)	6.3[b] [1.0 – 32.3] (n = 90)	5.5[b,c] [1.8 – 15.2) (n = 55)	3.9[c] [1.5 – 9.2] (n = 31)
Survivors	9.7*[a] [1.5 – 38.6] (n = 104)	6.0[b] [1.0 – 26.4] (n = 79)	5.5[b] [1.8 – 15.2] (n = 50)	3.3[c] [1.5 – 9.2] (n = 27)
Non-survivors	15.8*[a] [3.2 – 55.9] (n = 45)	10.1 [4.1 – 32.3] (n = 11)	5.3[b] [3.4 – 9.5] (n = 5)	6.3 [3.9 – 9.2] (n = 4)
ACTH/Cortisol				
Total population	2.2 [0.1 – 66.6] (n = 146)	2.3 [0.1 – 50.4] (n = 83)	2.3 [0.3 – 13.2] (n = 50)	2.7 [0.5 – 13.3] (n = 28)
Survivors	1.8* [0.1 – 66.6] (n = 101)	2.2 [0.1 – 50.4] (n = 72)	2.3 [0.3 – 6.8] (n = 45)	2.7 [0.5 – 13.3] (n = 24)
Non-survivors	3.8* [0.8 – 32.6] (n = 45)	2.3 [1.0 – 12.2] (n = 11)	2.3 [1.4 – 13.2] (n = 5)	2.8 [1.1 – 9.3] (n = 4)

Differences were determined using a mixed effects model; with different letters indicate a significant difference between days ($P < 0.05$)
* indicates a significant difference difference between survivors and non-survivors for ACTH ($P <$ .0009), cortisol (P = .012) and ACTH/cortisol ratio (P = .0001).

Supplementary data Table 2. Changes in the median [range] plasma ACTH and serum cortisol concentrations and the ACTH/cortisol for horses with and without SIRS over 6 days of hospitalization.

	Admission	Day 2	Day 4	Day 6
ACTH (pg/mL)				
No SIRS	16.6[a] [0.9 – 775.1] (n = 73)	12.7 [0.5 – 131.6] (n = 44)	11.9 [2.4 – 68.5] (n = 23)	6.7[b] [0.9 – 42.5] (n = 15)
SIRS	35.0[a] [3.6 – 449.8] (n = 75)	15.4[b] [3.6 – 278.0] (n = 40)	12.4[b] [6.1 – 47.2] (n = 27)	10.2[b] [3.9 – 49.9] (n = 14)
Cortisol (μg/dL)				
No SIRS	10.5[a] [3.3 – 51.3] n = 74	6.3[b] [1.0 – 32.3] (n = 49)	5.5[b,c] [3.2 – 10.9] (n = 26)	4.3[c] [1.7 – 7.0] (n = 15)
SIRS	11.8[a] [1.5 – 55.9] (n = 75)	6.0[b] [2.3 – 19.0] (n = 41)	5.5[c] [1.8 – 15.2] (n = 29)	3.3[d] [1.5 – 9.2] (n = 16)
ACTH/Cortisol				
No SIRS	1.8 [0.1 – 66.6] (n = 72)	2.4 [0.1 – 12.2] (n = 43)	2.2 [0.3 – 7.2] (n = 23)	1.6 [0.5 – 9.3] (n = 14)
SIRS	2.9 [0.5 – 33.4] (n = 74)	2.2 [0.5 – 50.4] (n = 40)	2.3 [0.5– 13.2] (n = 27)	3.2 [1.1 – 13.3] (n = 14)

The presence of SIRS is defined as having ≥ 2 SIRS criteria which are defined as: heart rate >52 bpm, respiratory rate >20 bpm, temperature below or above 37.0–38.5°C, WBC below or above 5.0–12.5x10^9/L (or presence of band neutrophils).
Differences were determined using a mixed effects model, with different letters indicate a significant difference between days ($P < .05$)

Supplementary data Table 3. Changes in the median [range] plasma ACTH and serum cortisol concentrations and the ACTH/cortisol ratio for SIRS score categories (0-4) over 6 days of hospitalization.

	Admission	Day 2	Day 4	Day 6
ACTH (pg/mL)				
SIRS 0	15.7 [0.9 – 158.4] (n = 27)	13.9 [1.3 – 35.2] (n = 19)	10.3 [2.4 – 51.9] (n = 8)	13.1 [6.7 – 27.6] (n = 5)
SIRS 1	19.0[a,c] [2.0 – 775.1] (n = 46)	11.2[a] [0.5 – 131.6] (n = 25)	13.8[b,c] [2.9 – 68.5] (n = 15)	4.0[b] [0.9 – 42.5] (n = 10)
SIRS 2	29.5 [3.6 – 398.9] (n = 43)	15.7 [3.6 – 139.0] (n = 27)	10.1 [6.1 – 47.2] (n = 17)	10.3 [3.9 – 49.9] (n = 9)
SIRS 3	36.4 [5.8 – 449.8] (n = 21)	15.1 [5.2 – 278.0] (n = 9)	15.0 [6.5 – 28.4] (n = 7)	16.6 [9.3 – 23.9] (n = 2)
SIRS 4	58.1 [14.4 – 123.4] (n = 11)	16.3 [12.3 – 36.0] (n = 4)	12.4 [6.4 – 22.2] (n = 3)	9.0 [5.5 – 20.6] (n = 3)
Cortisol (µg/dL)				
SIRS 0	10.5[a] [5.1 – 32.0] (n = 27)	6.4[b] [1.0 – 18.4] (n = 21)	5.0[b] [3.2 – 9.4] (n = 9)	5.5[b] [4.2 – 8.7] (n = 4)
SIRS 1	10.5[a] [3.3 – 51.3] (n = 47)	6.3[b] [2.5 – 32.3] (n = 28)	5.7[b] [3.4 – 10.9] (n = 17)	4.1[c] [1.7 – 5.5] (n = 11)
SIRS 2	10.6[a] [1.5 – 55.9] (n = 44)	6.8[b] [2.3 – 18.9] (n = 28)	5.8[b] [2.3 – 15.2] (n = 18)	4.7[b] [2.5 – 9.1] (n = 10)
SIRS 3	11.9[a] [3.2 – 24.5] (n = 20)	5.4[b] [4.1 – 9.5] (n = 9)	5.0[b] [3.1 – 13.7] (n = 8)	3.3 [2.9 – 9.2] (n = 3)
SIRS 4	16.7 [3.9 – 20.0] (n = 11)	2.4 [2.3 – 8.4] (n = 4)	3.7 [1.8 – 5.3] (n = 3)	1.6 [1.5 – 3.3] (n = 3)
ACTH/Cortisol				
SIRS 0	1.5 [0.1 – 11.9] (n = 27)	2.6 [0.3 – 4.8] (n = 18)	1.9 [0.5 – 6.7] (n = 8)	2.3 [1.5 – 4.1] (n = 4)
SIRS 1	2.2 [0.2 – 66.6] (n = 45)	2.1 [0.1 – 12.2] (n = 25)	2.2 [0.3 – 7.2] (n = 15)	1.3 [0.5 – 9.3] (n = 10)
SIRS 2	2.4 [0.5 – 33.8] (n = 43)	2.1 [0.5 – 20.0] (n = 27)	2.2 [0.6 – 13.2] (n = 17)	2.9 [1.1 – 9.1] (n = 9)
SIRS 3	3.3 [1.1 – 18.4] (n = 20)	2.3 [1.0 – 50.4] (n = 9)	2.6 [0.5 – 6.8] (n = 7)	5.3 [3.2– 7.3] (n = 2)
SIRS 4	3.9 [0.7 – 14.9]	6.7 [1.6 – 15.5]	3.6 [2.3 – 6.0]	3.7 [2.8 – 13.3]

	(n = 11)	(n = 4)	(n = 3)	(n = 3)

The SIRS score is based on the number of abnormal SIRS criteria based on: heart rate >52 bpm, respiratory rate >20 bpm, temperature below or above 37.0–38.5°C, WBC below or above 5.0–12.5x10^9/L.

Differences were determined using a mixed effects model, with different letters indicating a significant difference between days ($P < .05$)

Supplementary data Table 4. Changes in the median [range] plasma ACTH and serum cortisol concentrations and the ACTH/cortisol ratio for horses with no SIRS (0-1 abnormal criteria); SIRS2: 2 abnormal SIRS criteria; SIRS3/4: 3 or 4 abnormal SIRS criteria over 6 days of hospitalization.

	Admission	Day 2	Day 4	Day 6
ACTH (pg/mL)				
SIRS 0/1	16.6[a] [0.9 – 775.1] (n = 73)	12.7 [0.5 – 131.6] (n = 44)	11.9 [2.4 – 68.5] (n = 23)	6.7[b] [0.9 – 42.5] (n = 15)
SIRS 2	29.5 [3.6 – 398.9] (n = 43)	15.7 [3.6 – 139.0] (n = 27)	10.1 [6.1 – 47.2] (n = 17)	10.3 [3.9 – 49.9] (n = 9)
SIRS 3/4	50.5[a] [5.8 – 449.8] (n = 32)	15.1 [5.2 – 278.0] (n = 13)	13.7[b] [6.4– 28.4] (n = 10)	9.3 [5.5 – 23.9] (n = 5)
Cortisol (µg/dL)				
SIRS 0/1	10.5[a] [3.3 – 51.3] (n = 74)	6.3[b] [1.0 – 32.3] (n = 49)	5.5[c] [3.2 – 10.9] (n = 26)	4.3[d] [1.7 – 8.7] (n = 15)
SIRS 2	10.6[a] [1.5 – 55.9] (n = 44)	6.8[b] [2.3 – 18.9] (n = 28)	5.8[b] [2.3 – 15.2] (n = 18)	4.7[b] [2.5 – 9.1] (n = 10)
SIRS 3/4	15.8[a] [3.2 – 24.5] (n = 31)	5.0[b] [2.3 – 9.4] (n = 13)	4.5[b] [1.8 – 13.7] (n = 11)	3.1[b] [1.5 – 9.2] (n = 6)
ACTH/Cortisol				
SIRS 0/1	1.8 [0.1 – 66.6] (n = 72)	2.4 [0.1 – 12.2] (n = 43)	2.2 [0.3 – 7.2] (n = 23)	1.6 [0.5 – 9.3] (n = 14)
SIRS 2	2.4 [0.5 – 33.8] (n = 43)	2.1 [0.5 – 20.0] (n = 27)	2.2 [0.6 – 13.2] (n = 17)	2.9 [1.1 – 9.1] (n = 9)
SIRS 3/4	3.9 [0.7 – 18.4] (n = 31)	2.7 [1.0 – 50.4] (n = 13)	2.6 [0.5 – 6.8] (n = 10)	3.7 [2.8 – 13.3] (n = 5)

The SIRS criteria are defined as: heart rate >52 bpm, respiratory rate >20 bpm, temperature below or above 37.0–38.5°C, WBC below or above 5.0–12.5x10^9/L.

Differences were determined using a mixed effects model, with different letters indicating significant differences between days (P < .05).

Supplementary data Table 5. Changes in the median [range] plasma ACTH and serum cortisol concentrations and the ACTH/cortisol for horses with and without an ischemic gastrointestinal lesion over 6 days of hospitalization.

	Admission	Day 2	Day 4	Day 6
ACTH (pg/mL)				
Non-ischemic lesion	17.0*[a,c]<br>[0.9 – 240.7]<br>(n = 97)	11.6[a,b]<br>[0.5 – 278.0]<br>(n = 61)	8.7[b,c]<br>[2.9 – 28.4]<br>(n = 31)	6.7[c]<br>[1.6 – 34.3]<br>(n = 15)
Ischemic lesion	57.0*[a]<br>[4.5 – 775.1]<br>(n = 51)	19.3<br>[7.1 – 139.0]<br>(n = 23)	16.6<br>[2.4 – 68.5]<br>(n = 19)	19.2[b]<br>[0.9 – 49.9]<br>(n = 14)
Cortisol (µg/dL)				
Non-ischemic lesion	9.4*[a]<br>[1.5 – 38.6]<br>(n = 98)	5.9[b]<br>[1.0 – 26.4]<br>(n = 67)	5.5[b]<br>[2.3 – 15.2]<br>(n = 35)	3.3[c]<br>[1.7 – 9.2]<br>(n = 16)
Ischemic lesion	15.2*[a]<br>[5.2 – 55.9]<br>(n = 51)	7.1[b]<br>[5.2 – 32.3]<br>(n = 23)	5.5[c]<br>[1.8 – 11.0]<br>(n = 20)	4.9[c]<br>[1.5 – 8.7]<br>(n = 14)
ACTH/Cortisol				
Non-ischemic lesion	2.0<br>[0.1 – 27.4]<br>(n = 95)	2.0<br>[0.1 – 50.4]<br>(n = 60)	1.6<br>[0.3 – 6.8]<br>(n = 31)	1.6<br>[0.7 – 7.1]<br>(n = 14)
Ischemic lesion	3.7<br>[0.5 – 66.6]<br>(n = 51)	2.8<br>[1.0 – 12.2]<br>(n = 23)	4.0<br>[0.5– 13.2]<br>(n = 19)	3.6<br>[0.4 – 13.3]<br>(n = 14)

Differences were determined using a mixed effects model, with a different letter indicating a significant difference between days ($P < .05$)

* indicates a significant difference between ischemic and non-ischemic lesions, $P < .0016$ for ACTH and $P = .0009$ for cortisol.
